# An analysis of national action plans on antimicrobial resistance in Southeast Asia using a governance framework approach

**DOI:** 10.1016/j.lanwpc.2020.100084

**Published:** 2021-01-23

**Authors:** Alvin Qijia Chua, Monica Verma, Li Yang Hsu, Helena Legido-Quigley

**Affiliations:** a Saw Swee Hock School of Public Health, National University of Singapore, Tahir Foundation Building, 12 Science Drive 2, #10-01, Singapore 117549, Singapore; b London School of Hygiene and Tropical Medicine, WC1H 9SH, London, UK

**Keywords:** Antimicrobial resistance, Global health, Health policy, Southeast Asia, Governance

## Abstract

The complex problem of antimicrobial resistance (AMR) is spread across human health, animal health, and the environment. The Global Action Plan (GAP) on AMR and context-specific national action plans (NAPs) were developed to combat this problem. To date, there is no systematic content analysis of NAPs from countries of the Association of Southeast Asia Nations (ASEAN). As the validity periods of most NAPs are ending, an analysis now will provide an opportunity to improve subsequent iterations of these NAPs. We analysed the current NAPs of ten ASEAN countries. We explored their objective alignment with GAP and performed content analysis using an AMR governance framework. Themes were broadly classified under five governance areas: policy design, implementation tools, monitoring and evaluation, sustainability, and One Health engagement. We identified policy priorities, useful features of NAPs, and specific areas that should be strengthened, including accountability, sustained engagement, equity, behavioural economics, sustainability plans and transparency, international collaboration, as well as integration of the environmental sector. Enhancement of these areas and adoption of best practices will drive improved policy formulation and its translation into effective implementation.

## 1 Introduction

Antimicrobial resistance (AMR) is a serious global public health threat. Inability to achieve returns on research and development (R&D) investments has resulted in ‘market failure’ for development of new antimicrobials [1]. New economic models may stimulate antimicrobial development, but this alone is insufficient. AMR, although both evolutionary and inevitable, is accelerated by many interrelated factors, which span across human health, animal health, and the environment [2,3]. As such, multisectoral collaborations across governments at national, regional, and global levels have been deemed necessary [4], [5], [6]. To address the multifaceted AMR problem, the World Health Organization (WHO) endorsed a Global Action Plan (GAP) in 2015, urging member states to develop their own context-specific, One Health approach-based, national action plans (NAPs) on AMR [7]. By 2018, over 100 countries developed their NAPs based on GAP, while 67 had initiated the process [8].

AMR burden is disproportionately high in low- and middle-income countries, due to reasons including sub-optimal health and food safety systems, as well as water and sanitation infrastructure [3,5] Tackling it requires a One Health approach to balance between ensuring equitable access to antimicrobials while limiting non-judicious usage [3]. A region of particular concern is Southeast Asia (SEA), defined in this paper as countries of the Association of Southeast Asia Nations (ASEAN), including Brunei, Cambodia, Indonesia, Laos, Malaysia, Myanmar, the Philippines, Singapore, Thailand, and Vietnam. SEA, an epicentre for emerging infectious diseases, has surfaced as a major AMR reservoir [9]. The region has recently undergone significant socioeconomic development and population growth, resulting in rapid augmentation of food-production systems and increased antimicrobial demand [10,11]. Irrational antimicrobial use (AMU), together with lack of awareness, weak infection prevention and control (IPC), unregulated access, self-medication, inadequate training among community health personnel, and low-quality or counterfeit drugs were some problems highlighted in SEA [11], [12], [13]. While political will has expedited NAP development in SEA, the progress on implementation, however, remains sub-optimal. During an ASEAN summit held in 2017, it was acknowledged that activities against AMR were still inadequate, and multisectoral collaborations were required [14]. During the summit, ASEAN leaders made a declaration to combat AMR through a One Health approach, to strengthen execution of AMR control activities, actively engage relevant stakeholders, develop defined objectives, and monitoring and evaluation (M&E) mechanisms.

The national policy action for AMR demands effective governance to mitigate its emergence and spread [15]. Governance refers to the manner in which decisions are made and implemented to respond to challenges or attain goals [16]. From a health systems perspective, WHO defines governance as ‘a wide range of steering and rule-making related functions carried out by governments/decision makers as they seek to achieve national health policy objectives’ [17]. It is the responsibility of governments to ensure that goals are clearly defined and adequate measures are taken to create conducive systems for their attainment. Emphasising on diffusion of power among multiple stakeholders, Kickbusch and Gleicher argued that whole-of-government and whole-of-society approaches are necessary in addressing complex and wicked problems [18]. In recent years, the governance paradigm has shifted from an authoritarian to a more collaborative one, enabling network mobilisation of each stakeholder to steer action [19].

### 1.1 Issues in governance of AMR

AMR governance requires collaborative approaches supported by a wide range of actors and multiple levels of governance [18]. The Interagency Coordination Group (IACG) on AMR recognised that stronger and sustained global leadership, advocacy, and a more powerful global narrative and vision were needed to advance global response to AMR [20]. Stakeholders under One Health approach include, policy/decision makers, regulatory authorities, medical and veterinary practitioners, pharmacists, representatives from pharmaceutical and animal feed industries, and environmental health [21]. AMR is a contested space where actors with distinct values present their version of the problem, resulting in different possible solutions [22]. Evidence shows that while endorsement from WHO created an initial momentum for development of AMR related call-to-action documents, collaborations were hard to sustain, for they were reliant on voluntary partnerships [23]. Lack of robust evidence regarding the political and economic context for coordinated and effective policy development has further complicated AMR policy formulation and implementation [24].

There is a paucity of research on evaluation of governance strategies and NAP assessment. The European Public Health Alliance undertook a study in 31 European countries, providing an overview of NAPs and challenges faced in their development and implementation [25]. Another study involving countries from the Western Pacific and SEA regions focused on how NAPs compared against GAP [26]. A framework on AMR governance for NAPs was published in 2019, but to our knowledge, no formal analysis using this framework has been conducted to date [27]. Therefore, the aim of our study is to assess NAPs from SEA and compare governance strategies adopted, which will help identify policy priorities for addressing AMR and best practices in policy formulation in the region. As most NAPs are nearing the end of their validity periods, an assessment now will provide an opportunity to improve subsequent iterations of these NAPs. Furthermore, since COVID-19 has claimed significant attention and national resources, we hope that this assessment will put AMR containment back on the map of essential programmes that cannot be neglected.

### 1.2 Conceptual framework of analysis

**Fig. 1:**
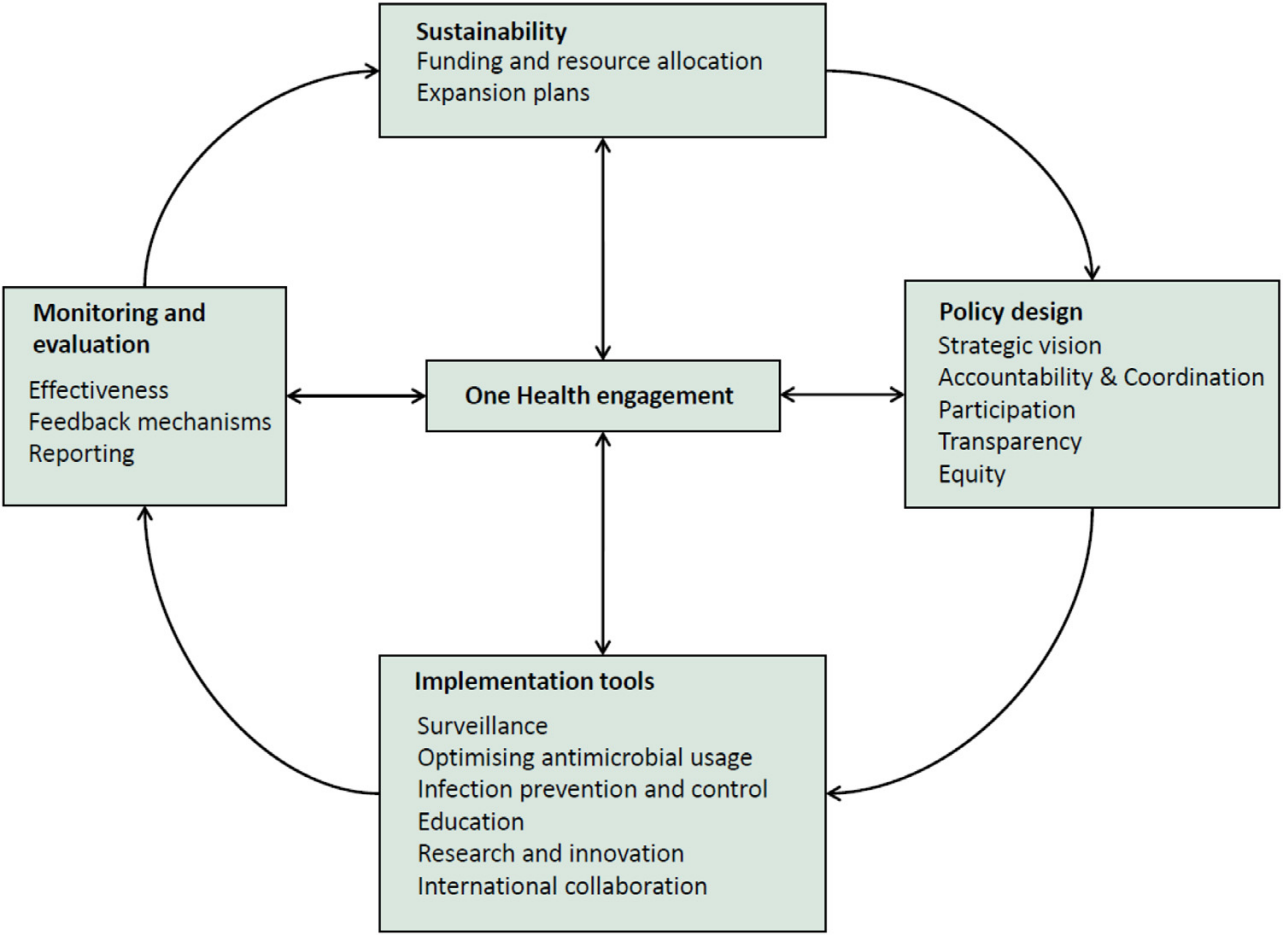
Framework for assessment of national action plans. *Source:* Amended from Anderson et al. 2019.

The complex nature of AMR demands a comprehensive framework to assess NAP governance. We used Anderson's framework to guide our evaluation [27]. The framework incorporates domains listed in WHO's situational analysis tool [28], and accounts for M&E. Spread across three governance areas including policy design, implementation tools, and M&E, the framework was conceptualised as a cyclical design for constant adjustments and improvements. During our analysis, we modified the framework, incorporating domains not featured in Anderson's framework and re-grouping existing ones that appropriately fit together. We identified an additional domain, ‘international collaboration’, and a few subdomains under ‘surveillance’ including: developing alert mechanisms for early detection and reporting of newly emerged resistance, hospital-acquired infection (HAI) surveillance, surveillance of food products for antibiotic residues, surveillance networks at the regional and national levels, and integration for better information sharing. Some domains including ‘public awareness’ and ‘education’, ‘antimicrobial stewardship’ and ‘medicines regulation’, as well as ‘AMR research’ and ‘fostering R&D’ have been regrouped. Drawing from WHO's situational analysis tool, we have situated One Health engagement at the centre of all governance areas [28]. Fig. 1 shows our updated framework.

## 2 Methodology

We included ten NAPs from SEA, obtained from WHO's library of NAPs [29], [30], [31], [32], [33], [34], [35], [36], [37], [38], [39]. NAP content analysis was performed using a two-pronged approach. First, we explored their alignment with GAP by comparing main objectives of each NAP against those in GAP. Second, we analysed their content using the AMR governance framework. Analysis was performed by two researchers (AQC and MV). We coded NAP contents deductively, with guidance from Anderson's framework, using line by line analysis during the initial round of independent coding. Inductive coding was also performed, allowing new codes to surface. Coding was conducted in an iterative manner, where we constantly reassessed documents coded initially and compared emerging codes. To increase process reliability, the team discussed codes and came to a consensus on the final list of themes. QSR NVivo 12 was used to organise and share data among team members.

## 3 Results

### 3.1 Alignment of NAPs with GAP on AMR

A summary of NAPs analysed is provided in Table 1. Specific validity periods were assigned for most NAPs. The One Health concept was reflected through involvement of multisectoral stakeholders in the design and implementation in all NAPs.

**Table 1:** Description of the national action plans from Southeast Asia.

Country	Name of document	Year	Purpose of policy	Stakeholders involved
Brunei	Antimicrobial Resistance National Action Plan	2019–2023	To serve as a strategy document to guide key stakeholders on the implementation of priority initiatives plans that requires urgent attention and are impactful in combatting antimicrobial resistance (AMR)	Ministry of Health, Ministry of Primary Resources and Tourism, as well as other organisations including a tertiary institution and a few hospitals.
Cambodia	Multi-Sectoral Action Plan on Antimicrobial Resistance in Cambodia 2019-2023	2019–2023	To guide the Royal Government of Cambodia, partners, and donors as they identify priority areas for work and collaboration.	Ministry of Agriculture, Forestry and Fisheries, Ministry of Health, Ministry of Environment, Ministry of Rural Development, medical facilities, tertiary institutions (both human and animal health),and laboratories, world health organization (WHO), Food and Agriculture Organization (FAO), OIE World Organisation for Animal Health, bilateral agencies, development banks and international/national Non-governmental organisations (NGOs).
Indonesia	National Action Plan on Antimicrobial Resistance Indonesia 2017-2019	2017–2019	To reflect the five principles based on which the GAP on AMR strategies have been enunciated.	Ministry of Health, Ministry of Agriculture, Ministry of Research Technology and Higher Education, Ministry of Marine Affair and Fisheries, Ministry of Defence, Minister of Foreign Affairs, Ministry of Information and Communication, hospital and professional associations, academics, and pharmaceutical industries, WHO, and FAO.
Laos	National Strategic Plan on Antimicrobial Resistance in Lao PDR 2019-2023	2019–2023	To develop the policy and cooperation mechanism under the framework of One Health to tackle the issue of AMR, and to develop and improve the capability of modern technology as it applies to the diagnosis and prevention of AMR.	Ministry of Health, Ministry of Agriculture and Forestry, academic institutions, hospitals. WHO, FAO and OIE provided technical and financial support.
Malaysia	Malaysian Action Plan on Antimicrobial Resistance (MyAP-AMR) 2017-2021	2017–2021	To slow the emergence of AMR and prevent its spread through four priority areas.	Ministry of Health, Ministry of Agriculture and Agro-based Industry, Ministry of Higher Education, Ministry of Defence Hospitals, private healthcare facilities, community pharmacists, the Animal Food Industry, professional organisations, academic institutions, the private sector, international partners, NGOs, and civil society.
Myanmar	Myanmar National Action Plan for Containment of Antimicrobial Resistance	2017–2022	To reflect the five principles based on which the Global Action Plan on AMR strategies have been enunciated.	Ministry of Health and Sports, Ministry of Agriculture, Livestock and Irrigation, Ministry of Education, Ministry of Commerce, Ministry of Home Affairs, Ministry of Defence, professional associations, WHO, FAO, OIE, and NGOs.
The Philippines	The Philippine Action Plan to Combat Antimicrobial Resistance - One Health Approach	2015–2020	To implement an integrated, comprehensive, and sustainable national program to combat AMR geared.	Department of Health, Department of Agriculture, Department of Science and Technology, Department of the Interior and Local Government, Department of Trade and Industry, local government units and the private sector.
Singapore	National Strategic Action Plan on AMR	2017	To unify and formalise the existing response mounted across animal, human, food, and environment sectors, while providing a roadmap to address existing gaps and prioritise future interventions.	Ministry of Health, Agri-Food & Veterinary Authority, the National Environment Agency, and PUB, the National Water Agency.
Thailand	Thailand's National Strategic Plan on AMR 2017-2021	2017–2021	To reduce the morbidity, mortality and economic burden caused by AMR, by establishing policies and national multi-sectoral mechanisms which support an effective and sustained AMR management system.	Ministry of Public Health, Ministry of Agriculture and Cooperatives, academics, professional societies, and civil society organisations.
Vietnam	National Action Plan for Combating Drug Resistance	2013–2020	To promote prevention of drug resistance, contributing to improving the quality and effectiveness of the prevention and control of epidemics, medical examination, and treatment to protect, care for and improve people's health.	Ministry of Health, Ministry of Agriculture and Rural Development.

AMR = Antimicrobial Resistance. WHO = World health Organization. FAO = Food and Agriculture Organization. NGO = Non-governmental organisation.

Table 2 highlights the objectives of NAPs and GAP. Each NAP had four to seven objectives, most of which were aligned with those in GAP. Four objectives in GAP were described in all NAPs. The objective on developing the economic case for sustainable investment, was less commonly discussed. It was absent from NAPs of Brunei and Malaysia, but was briefly mentioned under the objective of 'Governance and coordination to reduce AMR’, the section on ‘international collaboration’, and ‘finance, scientific research, and international collaboration’ in the NAPs of Cambodia, Singapore, and Vietnam respectively. Some NAPs mentioned objectives which were not listed in GAP. Cambodia and Laos mentioned governance and coordination between stakeholders to combat AMR, while the Philippines, Thailand, and Vietnam highlighted ensuring uninterrupted supply of quality medicines.

**Table 2:** Comparison of objectives in each national action plan against objectives in the global action plan on antimicrobial resistance.

Document	Key objectives in line with the global action plan (GAP) on antimicrobial resistance (AMR)					Objectives not in GAP
GAP	Improve awareness and understanding of AMR through effective communication, education, and training	Strengthen the knowledge and evidence base through surveillance and research	Reduce the incidence of infection through effective sanitation, hygiene, and infection prevention measures	Optimise the use of antimicrobial medicines in human and animal health	Develop the economic case for sustainable investment, and increase investment in new medicines, diagnostic tools, vaccines, and other interventions	..
Brunei	Awareness and education	Surveillance and research	Infection prevention and control	Optimise use of antimicrobials	..	..
Cambodia	Increasing public awareness; Building human capacity for AMR	Evidence generation through surveillance and laboratories; Research and innovation for AMR	Containing AMR through good practices	Rational use of antimicrobial medicines	..	Governance and coordination to reduce AMR
Indonesia	Improve awareness and understanding of AMR through effective communication, education, and training	Strengthen knowledge and evidence base through surveillance and research	Reduce incidence of infection with sanitation, hygiene, and infection prevention	Optimise use of antimicrobial medicines in human and animal health	Develop economic case for sustainable investment and increase investment in new medicines, diagnostic tools, vaccines, and other interventions	..
Laos	Improve awareness and understanding of AMR	Strengthen the AMR surveillance system	Improve infection prevention and control	Optimise the use of antimicrobial agents in humans and animals	Improve coordination and budget support	
Malaysia	Public awareness and education	Surveillance and research	Infection prevention and control	Appropriate use of antimicrobials	..	..
Myanmar	Improve awareness and understanding of AMR through effective communication, education, and training	Strengthen the knowledge and evidence base through surveillance and research	Reduce the incidence of infection through effective sanitation, hygiene, and infection prevention measures	Optimise the use of antimicrobial medicines in human and animal health	Develop the economic case for sustainable investment that takes account of the needs of all countries, and increase investment in new medicines, diagnostic tools, vaccines, and other interventions	..
The Philippines	Development of a risk communication plan to combat AMR	Strengthen surveillance and laboratory capacity; Foster innovation, research, and development	Enhance infection prevention and control across all settings	Regulate and promote the rational use of medicines in the human and animal health sectors	Commit to a comprehensive, financed national plan with accountability and civic society engagement	Ensure uninterrupted access to essential medicines of assured quality
Singapore	Education	Surveillance and risk assessment; Research	Prevention and control of infection	Optimisation of antimicrobial use	..	..
Thailand	Public knowledge on AMR and awareness of appropriate use of antimicrobials	AMR surveillance system using a 'One-Health' approach	Infection prevention and control and antimicrobial stewardship in humans; AMR prevention and control and antimicrobial stewardship in agriculture and animals		Governance mechanisms to develop and sustain AMR-related actions	Regulation of antimicrobial distribution
Vietnam	Raise awareness of community and health workers on drug resistance	Strengthen, improve national surveillance system on the use of antibiotics and drug resistance	Promote infection control	Promote proper safe use of drugs; Promote proper safe antibiotic use in livestock, poultry, aquaculture, and cultivation	..	Ensure adequate supply of quality medicines to meet the needs of people.

GAP = Global Action Plan. AMR = Antimicrobial resistance.

### 3.2 Assessment of NAPs

Using the adapted conceptual framework, we assessed five governance areas, including policy design, implementation tools, M&E, sustainability, and One Health engagement.

#### 3.2.1 Policy design

Policy design involves fundamental procedures required for effective NAP implementation. Features of each NAP based on domains in this governance area are presented in Table 3. Some NAPs briefly mentioned ongoing activities to combat AMR, while describing upcoming planned activities under each objective.

**Table 3:** Governance area 1 – Policy design.

Country	Strategic vision	Accountability & coordination	Participation	Transparency	Equity
Brunei	• Situational analysis conducted: January - March 2019. • No mention of specific, measurable, achievable, relevant, and time-bound (SMART) targets. • Tools like self-assessment surveys used to identify strengths and challenges of current system.	• The multi-sectoral Brunei Darussalam Antimicrobial Resistance Committee (BDAMRC) of the Ministry of Health and the Ministry of Primary Resources and Tourism were in charge. • Four technical working groups (TWGs) responsible for each individual strategic objective.	• Periodic stakeholder and community engagement activities such as workshops conducted prior to the development of the national action plan (NAP) and during implementation to identify strengths and challenges of existing systems.	• BDAMRC was projected as a platform for information sharing, notification, and communication regarding antimicrobial resistance (AMR) related issues.	• No mention of equitable access to existing essential antimicrobials. • Research to guide considerations for socioeconomic burden in the population was mentioned.
Cambodia	• Situational analysis conducted: October - December 2017. • SMART objectives mentioned, such as: • 10-20% ↓ of irrational antimicrobial use (AMU) in humans and animals • 10-20% ↓ of hospital-acquired infections • Regular baseline assessment to be conducted to identify gaps across human, animal, livestock, and environment.	• The TWG on AMR of the Ministry of Health and the Ministry of Agriculture, Forestry and Fisheries, and Ministry of Environment were in charge. • Lead institutions were assigned for every activity. • Effective governance coordination emphasised in Strategic Objective 1.	• Relevant stakeholders engaged in NAP development to review the draft, validate results, confirm gaps, and endorse the strategic focus areas. • Continuous engagement planned for regular TWG meetings for coordination and information sharing with stakeholders. • Partnerships strengthening with professional associations and political leaders emphasised.	• An overarching research agreement to be implemented, covering all sectors and relevant ministries to ensure optimal efficiency, transparency, and data sharing.	• Equitable access to antimicrobials included in Strategic Objective 3. • Measures including strengthening supply chain management for antimicrobials, publishing essential medicine list and clinical guidelines mentioned to ensure equitable and universal access in human health, animal health and agriculture.
Indonesia	• Situational analysis conducted: May 2016. • SMART indicators for each objective mentioned, such as: • Number of quality assured laboratories supporting AMR surveillance sites • Number of revised curricula for target professional groups	• The Inter-Ministerial Steering Committee of the Ministry of Health, Ministry of Defence, Ministry of Agriculture, and Ministry of Foreign Affairs were in charge. • The National AMR Coordination Committee (NARCC) assisted in planning and implementation through five TWGs for each objective. • Responsible agencies were assigned for every activity.	• Multi-sectoral stakeholders across human, animal, and environment sectors constituting NARCC ensured integration of AMR containment efforts in the system.	• Data and information unit to be created to store AMR information for utilisation by government agencies, the general public, and international community as appropriate. • National Drug Policy on antimicrobials available in the public domain to be formulated. • Data on AMU and sales in humans, animals, and fisheries to be made publicly available	• Regulation of post-marketing quality of drugs to ensure access to safe and quality antibiotics.
Laos	• Situational analysis conducted: 2015 • SMART targets to be achieved by 2023 mentioned, such as: • 30% ↓ in number of resistant infections • 80% of consumers to be educated on AMR by doctors and veterinarians • 20% ↑ in awareness levels	• The AMR Surveillance and Control Committee was in charge. • Two sub-committees, consisting of multi-sectoral representatives were responsible. • Improving coordination included in Strategic Objective 5.	• A strategic plan development committee was set up to draft the NAP. • Sensitisation of policymakers on policies related to AMR ensured through advocacy efforts. • Continuous engagement of multi-sectoral collaboration ensured throughout the implementation at all levels through regular monthly meetings.	• No mention of public access to AMR and AMU surveillance data, progress reports, and funding information.	• Strengthening of supply chain and logistic systems and expansion of centralised price negotiation procurement to increase availability of medicinal products highlighted. • Policy to promote appropriate AMU and minimise socioeconomic losses in society was highlighted.
Malaysia	• No formal situational analysis conducted. Existing data from ongoing surveillance programmes highlighted. • SMART evaluation indices mentioned for each strategy, such as: • Number of AMR awareness activities/campaign conducted • Number of medical institutions participating in AMR surveillance	• The National AMR Committee (NARC) of the Ministry of Health and Ministry of Agriculture and Agro-based Industry were in charge. • The National Coordinating Centre for AMR coordinated activities through a supporting structure comprising of four TWGs for each objective. • Responsible agencies were assigned for every activity.	• All stakeholders in NARC were engaged in providing technical support and reviewing outcomes.	• No mention of public access to AMR and AMU surveillance data, progress reports, and funding information.	• No mention of equitable access to existing essential antimicrobials.
Myanmar	• Situational analysis conducted: September 2016. • SMART indicators stated for each objective, such as: • Number of quality assured laboratories supporting AMR surveillance sites • Number of revised curricula for target professional groups	• The National Multi-Sectoral Steering Committee of the Ministry for Health & Sports and Ministry for Agriculture, Livestock & Irrigation, were in charge. • The National AMR Coordinating Centre (NACC) assisted in planning and implementation through a supporting structure comprising of five TWGs for individual strategic objectives. • Responsible agencies were assigned for every activity.	• NACC organised regular bi-annual meetings. • All relevant stakeholders gave technical inputs for situation analysis.	• Central database to be established to store AMR risk information, and made available to government agencies, public and international community as appropriate. • National Drug Policy on antimicrobials and AMR in humans and animal health, as well as aquaculture to be formulated and made available in the public domain.	• Pharmaceutical supply chain, including the procurement, supply and management system in human health, veterinary and food production sectors to ensure access to antimicrobials to be strengthened. • Research on socioeconomic burden of AMR was highlighted.
The Philippines	• Situational analysis conducted: 2012 • SMART targets mentioned, such as: • 30% ↓ in carbapenem-resistant Enterobacteriaceae and multidrug-resistant Pseudomonas spp. hospital-acquired infections • At least 30% ↓ in overall methicillin resistance in *Staphylococcus aureus* bloodstream infections.	• The Inter-Agency Committee on AMR of the Department of Health and Department of Agriculture were in charge. • Responsible agencies were assigned for every activity.	• Relevant stakeholders participated in a summit to lay out plans and strategies to combat AMR prior to NAP development.	• Risk communication plan to be implemented to provide relevant scientific information that is accessible to all and effectively understood by everyone. • Fund allocation was listed for each objective.	• Availability of new antibiotics for priority infectious diseases to be facilitated. • Issuance regulations on access to antimicrobials, particularly for the distribution and sale in drug outlets to be reviewed. • Highlighted socioeconomic status and its effect on patient compliance to drug administration.
Singapore	• No formal situational analysis conducted. • SMART targets are absent, to be detailed in the subsequent plan.	• The One Health AMR Workgroup of the Ministry of Health, the Agri-Food and Veterinary Authority, the National Environment Agency, and PUB, the National Water Agency, were in charge. • Multi-sectoral agencies coordinate for data integration on resistant patterns and sharing on a single platform.	• Need of coordinated action from a wide range of stakeholders, together with community engagement during implementation, was recognised.	• No mention of public access to AMR and AMU surveillance data, progress reports, and funding information.	• Access to safe and effective vaccines for disease prevention in the farming industry mentioned. • Research on socioeconomic burden of AMR was highlighted.
Thailand	• No formal situational analysis conducted. Situation of AMR in Thailand described in NAP. • SMART targets by 2021 mentioned, such as: • 20% ↓ in AMU in humans • 30% ↓ in AMU in animals	• The Coordinating and Integrating Committee on AMR of the Ministry of Public Health was in charge. • The National AMR Coordination Committee assists in planning and implementation through a supporting structure comprising of five TWGs for individual strategic objectives.	• Stakeholders actively participated in preparing the draft. • Ongoing regular meetings and workshops to identify strategic solutions and explore methods of collaboration.	• A technical report 'The Landscape of AMR Situations and Actions in Thailand' was made available to stakeholders to understand the overall AMR situation and the actions taken in Thailand, prior to NAP establishment. • Participatory conceptualisation and NAP development were detailed.	• Regulations on reclassification of antimicrobials to incorporate the factors associated with access to medicines and health care services.
Vietnam	• No formal situational analysis done. Situation of AMR in Vietnam described in NAP. • While no SMART targets are mentioned, timelines have been assigned for phases of implementation.	• The Steering Committee of the Ministry of Health and Ministry of agriculture and rural development were in charge. • Responsibilities were assigned to multi-sectoral agencies. • Focal authority was appointed to facilitate coordination for implementation	• Thrust on participation and collaboration was lacking.	• No mention of public access to AMR and AMU surveillance data, progress reports, and funding information.	• Prioritisation of production of generic drugs in the country and investment in production supply the market with drugs of good quality, reasonable price. to improve access and availability. • Essential medicines list to be updated according to the pattern of disease and socio-economic conditions.

SMART = Specific, Measurable, Achievable, Relevant and Time-bound. BDAMRC = Brunei Darussalam Antimicrobial Resistance Committee, TWG = Technical working group. NAP = National action plan. AMR = Antimicrobial resistance. AMU = Antimicrobial use. NARCC = National Antimicrobial Resistance Coordination Committee. NARC = National Antimicrobial Resistance Committee. NACC = National Antimicrobial Resistance Coordinating Centre.

##### 3.2.1.1 Strategic vision

Strategic vision entails the goals and objectives of NAPs. All countries, except Singapore, have conducted situational analyses to assess AMR burden locally and to inform strategies to mitigate AMR. Apart from Brunei, Singapore, and Vietnam, all countries discussed objectives which were specific, measurable, and timebound. Cambodia, Laos, Thailand, and the Philippines took a step further by defining specific quantitative targets for AMR and AMU in humans and animals.

##### 3.2.1.2 Accountability and coordination

Coordination is facilitated by accountability, wherein an individual or an institution has been assigned an appropriate responsibility with clearly defined targets, and are answerable to other authorities, with consequences if outcomes are not satisfactory [40,41]. Coordination between sectors and across different levels of each sector is important for a multi-sectoral One Health approach. This was present in all NAPs and was highlighted strongly by Cambodia as a key objective. The need for a ministry or intersectoral committee for coordination and implementation was mentioned in all NAPs, albeit at various degrees. Very often, Technical Working Groups (TWGs) were formed to work on specific subject matters. Accountability, although common across all NAPs through nomination of a responsible party, was rarely discussed in detail and implications of unmet objectives were absent. Accountability to authorities such as the Ministry of Health was mentioned in some NAPs.

##### 3.2.1.3 Participation

Wide stakeholder participation increases support for implementation and evaluation of containment measures. High level of stakeholder participation was facilitated throughout development of nine NAPs. On the contrary, Singapore shared very limited information on its NAP conceptualisation and implementation. Consistent engagement and strengthening support emerged as a weak concept, for only five NAPs stated periodic meetings and engagement activities.

##### 3.2.1.4 Transparency

Transparency, via availability of accurate and updated data, enables political awareness to mobilise support and improves public participation in the policy process, by gaining trust and bolstering collaboration. Six countries accounted for transparency by establishing platforms for data sharing, including data on AMU surveillance. The Philippines underpinned the need for effective communication at all levels for success and sustainability, and commitments to enhance coordination. Thailand detailed the process of its NAP conceptualisation and development.

##### 3.2.1.5 Equity

Equity, with regards to AMR, needs to be understood in terms of accessibility and affordability of healthcare and quality antimicrobials, as well as gender and socio-economic considerations where vulnerable groups may be at higher AMR exposure risks. All countries, except Brunei and Malaysia, accounted for equity, either directly by ensuring accessibility and affordability of quality antimicrobials or indirectly by maintaining uninterrupted supply chains. Singapore only mentioned about access to vaccines. Socio-economic considerations were accounted for in six countries, while gender equity was not mentioned by any country.

#### 3.2.2 Implementation tools

This governance area involves interventions to combat AMR across the One Health spectrum. Tables 4 and 5 highlight domains of this area.

**Table 4:** Governance area 2 – Implementation tools (part 1).

Country	Surveillance	Optimising antimicrobial usage	Infection prevention and control (IPC)
Brunei	Prior/ongoing activities • Data collection on antimicrobial consumption, including appropriateness of usage is ongoing. Future actions • Standardised reference laboratory to be setup and laboratory capacity strengthened. • National surveillance system for antimicrobial resistance (AMR) in human, animal, and environment health sectors to become functional. • Surveillance in public and private hospitals include point prevalence surveys (PPS) on hospital-acquired infections (HAIs) and AMU. • National monitoring system for antimicrobial use (AMU), integrating data from public and private healthcare sectors, and commercial farms and poultry slaughterhouses to be established. • Routine testing of the presence of antibiotic residues in food chain in pipeline.	**Antimicrobial Stewardship Programmes (ASPs)** Prior/ongoing activities • ASPs were in place in selected facilities in both human and animal sector. • Guidelines for best practices such as National Antibiotic Guidelines and National Guidelines for Prudent Use of Antimicrobials in Livestock, as well as the National Standard Drug List have been developed, and were continuously reviewed and updated. Future actions • Economic incentives that contribute towards inappropriate AMU to be analysed and introduced to encourage judicious AMU. **Regulations** Prior/ongoing activities • All antimicrobials were classified as prescription only medicines that can only be prescribed by registered professionals. Future actions • Pharmacovigilance Programme and Animal Feed Programme to strengthen regulations to restrict AMU as animal growth promoters and prophylaxis. • All medicinal products intended for human use, must be registered with the Brunei Darussalam Medicines Control Authority, to ensure all marketed medicinal products are safe, efficacious and of good quality. • Veterinary Surgeons Order to ensure veterinarians were licensed and abide by a code of ethics for prudent AMU to be enacted. • Poisons Order to monitor all imported veterinary medicinal products to be enacted.	**Policies and guidelines** Future actions • National IPC programme, including hand hygiene practices for human health, to be developed. • Programmes for Good Agriculture Practices, Good Animal Husbandry Practices and Veterinary Health Mark for animal health, to be developed. **Other strategies** Future actions • Human vaccination programmes to be strengthened; guidelines on vaccination requirements for all veterinarians and para vets practicing in the veterinary clinics to be developed. • Campaigns and programmes to educate public and professionals to be established.
Cambodia	Prior/ongoing activities • Strengthening of laboratories capacity. • Ongoing continuous surveillance of AMU in human and animal health and agriculture, including PPS for AMU appropriateness and HAI in humans. Future actions • Centralised laboratory database for AMR surveillance data to be established. • Joint AMR surveillance between human, animal, and environmental sectors to be strengthened to enhance understanding of cross-transmission; early detection mechanism for outbreaks to be implemented. • Antimicrobial residue monitoring in food, agriculture, and environment to be implemented. • Coordination of laboratory and surveillance activities through national and regional network to be supported. •	**ASP** Future actions • ASPs to be developed and implemented in target health facilities with supporting local policies, staffing, dedicated teams, and budgets. • Guidelines to be implemented in the animal and environmental sectors; AMR to be incorporated in the upcoming revision of Clinical Practice Guidelines, Essential Medicines List, and other guidelines for AMU. **Regulations** Future actions • Regulatory and legislative frameworks to be reviewed and strengthened in different aspects of AMU, surveillance and monitoring, including AMR response, poor quality medicines, prescription-only dispensing of antimicrobials, waste management, registration of facilities, registration of animal clinics and farms, licensing, AMU for animal disease prevention and growth promoters, and commercial feeds and maximum residue limits. • Services of pharmacies and health facilities to be strengthened through registration and licensing; sanitary and environmental regulations on farm practices for terrestrial and aquatic animals to be strengthened. • Institutional procurement and supply chain systems to be strengthened to ensure availability of quality antibiotics. • No clear mention of authority in place to monitor and enforce legislation.	**Policies and guidelines** Future actions • National policy and guidelines on IPC to be developed; roles and responsibilities of hospital IPC committees to be strengthened; availability of IPC infrastructure and supplies to be ensured. • Association of Southeast Asian Nations and international good agriculture practice guidelines to be adopted to ensure animal health; provision of IPC infrastructure and hygiene supplies and equipment to be improved in animal clinics, veterinary practices, slaughter facilities and live animal markets. • Infrastructure to be set up to support food safety inspection and analysis. • Best practice guidance to be developed for safe disposal of unused or expired antibiotics and animal feeds, alternatives to antimicrobial growth promoters, waste management, and water monitoring. **Other strategies** Future actions • Best practice guidance for vaccinations to be developed.
Indonesia	Future actions • Laboratory capacity to be enhanced; reference laboratory to be set up with standardised laboratory operations; standards and guidelines based on international standards to be developed. • National AMR surveillance programme to be implemented for human, animal, food, and aquaculture, including HAI surveillance; surveillance system to allow for early detection of resistance to be developed; surveillance to be extended to district and rural hospitals in public and sentinel private hospitals/chains of hospitals. • AMU monitoring programme in humans and food animals including residues testing in food products to be developed. • National laboratory surveillance network to be established; platform for seamless data transmission to be set up.	**ASP** Future actions • Comprehensive evidence-based ASPs and guidelines for national ASP in human, animal health care and aquaculture to be established in ambulatory and community settings; ASP to be added into existing hospital accreditation qualifying criteria. • Manuals on the use of antimicrobials, including an essential antibiotics list and standard treatment antimicrobial guidelines in human medicine, veterinary medicine, and aquaculture to be developed. **Regulations** Future actions • National AMR Containment and Use Policy and related regulatory frameworks to be developed to control the AMU in humans and animals. • Antimicrobial growth promoters to be phased out and certification system of farm products free from antibiotic residue to be established. • Independent periodic surveys to estimate the extent of non-prescription sales of antibiotics to be conducted. • Regulations for antimicrobials (veterinary, human, and aquaculture) and its import, manufacture, quality, distribution, market authorisation, advertising, and inspection, tracking and reporting, to be developed. • Regulations to be enforced by the Drug Regulatory Authority.	**Policies and guidelines** Future actions • National IPC programme to be established within healthcare settings, animal husbandry systems and fisheries and the food chain; IPC education for healthcare providers, veterinarians, and food handlers to be conducted. • IPC performance with hospital accreditation system for human health to be linked. • IPC biosecurity and vaccination in animal health facilities and farms to be developed, in line with international standards set out by Food and Agriculture Organization (FAO) and OIE, World Organisation for Animal Health. • Coordination with surveillance units in the field of agriculture and environmental health to assess AMR hazard and risk to be established. • Formal campaigns for sanitation and hygiene to be developed. **Other strategies** Future actions • Human vaccination programme to be strengthened; animal vaccination programmes and campaigns to be established.
Laos	Future actions • Standardised reference laboratory to be set up. • Laboratory capacity to be strengthened; biannual training on laboratory techniques and AMR surveillance. • AMR and AMU to be monitored, including appropriateness, in humans and animals, as well as residues in animal feeds and food products. • Centralised laboratory database for AMR surveillance data to be established.	**ASP** Future actions • Capacity building on the rational AMU and ASPs for medical doctors, veterinarians, and pharmacists to be provided. • Guidelines on AMU in the animal sector and standardised guideline for management of antibiotic-resistant pathogen in the human sector to be developed. **Regulations** Future actions • Legislation to be developed to control AMU in humans by declaring antimicrobials as a controlled medicine; monitoring of importation and distribution; sale of antimicrobials only by a qualified professional upon presentation of a prescription. • Regulations for AMU in agriculture and livestock; control the manufacturing, import and export of veterinary drugs and their distribution; regulation to control the production, sale and use of antibiotics in food and water for livestock. • The Food and Drug Law to cover the production/registration/control of quality pharmaceuticals to ensure the quality, efficacy, and safety of antimicrobials. • Food and Drug Department enforces laws and regulations on AMU.	**Policies and guidelines** Future actions • National strategic plan for IPC in healthcare facilities for human health, and at the national, central, and provincial levels to be developed. • IPC to be improved in government hospital wards. • Agricultural sector to be included at the national level as part of the purview of the IPC committee. • Waste management systems and sanitation plans in hospitals/farms to ensure safe disposal in environment to be set up.
Malaysia	Prior/ongoing activities • Standardised reference laboratory already present. • Ongoing AMU surveillance and HAI in selected hospitals since 2001; regular national PPS on antibiotic utilisation conducted in healthcare facilities. Future actions • Laboratory capacities to be enhanced and test methods standardised across laboratories; capacity of personnel to be improved. • An integrated surveillance on AMR and AMU involving human and animal health was underway; AMR surveillance in place and to be strengthened for animal health and aquaculture. • Alert mechanism for AMR detection and reporting of newly emerged resistance to be developed. • A networking/web-based system between the Ministry of Health, university, and private hospitals to be developed.	**ASP** Future actions • ASPs to be reinforced at the healthcare facilities with the establishment of ASP policies and teams as a criterion for hospital accreditation. • National Veterinary Antibiotic Guideline and list of controlled antibiotics for food producing animal to developed; National Essential Medicines Lists, National Antibiotic Guideline, and Clinical Pathway for Common Infections to be regularly reviewed. • Critically important antibiotics for human in veterinary/aquaculture use to be phased out. • Introduction of incentives to optimise appropriate use of antimicrobial agents. **Regulations** Future actions • Distribution, prescription & dispensing of antimicrobials in accordance with national legislation to be ensured; import quantity of specific antibiotics for human and veterinary usage to be monitored; regular inspections/audits planned on the sale of antimicrobial agents in community pharmacies • Regulation on prescription of antimicrobial in animal feed and usage in aquaculture; AMU report as a condition for license application or renewal for farmers. • Pharmacy Enforcement Division and Department of Veterinary Services regulates legislation in human and animal health, respectively.	**Policies and guidelines** Future actions • National policies and standards of practice on IPC to be strengthened in health facilities for human health, including hand hygiene programmes; National Policies and Procedures on Infection Control to be revised; bi-annual IPC audit and training to be conducted. • Improvement of biosecurity and husbandry for animal health, annual audits in licensed farms and processing plants to be conducted; surveillance and IPC practices according to standards from the OIE to be strengthened. **Other strategies** Future actions • Vaccination in human and food animals to be promoted; animal health programmes to be optimised by vaccination of pets; market access for products from farm with audit certifications to be facilitated.
Myanmar	Future actions • Reference laboratory to be set up and laboratory operations to be standardised. • National AMR surveillance programme to be implemented in central hospitals, and central and regional veterinary diagnostic laboratories; national early warning system to identify early emergence of resistance to be set up. • AMU monitoring programme in humans and food animals including residues testing in food products to be developed; PPS to assess quantity and quality of AMU in different settings; HAI surveillance in selected public and private healthcare facilities to be expanded further.	**ASP** Future actions • Comprehensive evidence-based ASPs and guidelines for national ASP in human, animal health and aquaculture to be established in ambulatory and community settings. • Manuals on AMU, including an essential antibiotics list and standard treatment antimicrobial guidelines in human medicine, veterinary medicine, aquaculture, and food production to be developed. **Regulations** Future actions • National AMR Containment and Use Policy and related regulatory frameworks to be developed to control the AMU in humans and animals. • Antimicrobial growth promoters to be phased out and certification system of farm products free from antibiotic residue to be established; independent periodic surveys to estimate the extent of non-prescription sales of antibiotics to be conducted. • Regulations for antimicrobials (veterinary, human, and aquaculture) and its import, manufacture, quality, distribution, market authorisation, advertising, and inspection, tracking and reporting, to be developed. • Regulations to be enforced by the Drug Regulatory Authority.	**Policies and guidelines** Future actions • National IPC programme to be established through compliance with the IPC guidelines within healthcare settings, animal husbandry systems and fisheries and the food chain; IPC education for healthcare providers, veterinarians and food handlers. • IPC performance with hospital accreditation system for human health to be linked. • IPC biosecurity and vaccination in animal health facilities and farms to be developed, in line with international standards set out by OIE/FAO; review of existing guidelines. • Coordination with surveillance units in the field of agriculture and environmental health to assess AMR hazard and risk; formal campaigns for sanitation and hygiene to be developed. **Other strategies** Future actions • Human vaccination programme to be strengthened; animal vaccination programmes and campaigns to be established.
The Philippines	Prior/ongoing activities • Standardised reference laboratory already present; • Sharing of data on emerging resistant pathogens with to AMR surveillance programme. Future actions • Laboratory facilities, equipment, and operations to be standardised and enhanced; trainings of human resources to be conducted. • Expansion of AMR surveillance programme to all hospitals retained by the Department of Health planned. • Integrated surveillance system for AMU, AMR and HAI in human health and livestock to be developed.	**ASP** Future actions • Development of ASPs in animal husbandry and in hospitals. • Essential medicines lists and national antibiotic guidelines for human and veterinary use to be developed; treatment guidelines for specific diseases to be updated; • Philippine Practice Standards for Pharmacists in relation to Rational Dispensing of Antimicrobials to be institutionalised. • Mainstreaming of complete treatment regimen through reimbursement schemes. **Regulations** Prior/ongoing activities • The Department of Health and Department of Agriculture strictly enforced regulations on antimicrobial manufacturing, promotion, marketing, prescription, dispensing, and use. Future actions • Issuances related to access (distribution and sales) to antimicrobials to be reviewed. • Database of registered antimicrobials, including quantitative production and importation, and monitoring of unregistered antimicrobials in animals planned • Safety, efficacy, and quality of medicines to be ensured from market authorisation to post-marketing surveillance for sustainable access to quality essential antimicrobials in human and animals.	**Policies and guidelines** Prior/ongoing activities • Good Animal Husbandry Practices and Good Aquaculture Practices for animal health has been adopted; the ‘Food Safety Act of 2013’ mandated responsibility for health of animals from where food was derived, and the effects of feeds and other production inputs on otherwise healthy animals. Future actions • National Policy on IPC for human health to be developed.
Singapore	Prior/ongoing activities • Food products and animal feed used at food farms are routinely tested for antibiotic residues. Future actions • National AMR reference laboratory to be established. • Standardised test methods and data reporting in alignment with international guidelines to be developed. • Routine HAI PPS surveillance to be established and extended to private hospitals and community settings. • AMR surveillance programme in animal health to be expanded to include all animal production sectors, namely poultry, ruminant and aquaculture farms. • Laboratory capacity for testing of AMR and antibiotic residues in food products to be reviewed. • Independent surveillance across the human, animal, food and environment sectors to be integrated to monitor spread between these sectors.	**ASP** Prior/ongoing activities • ASPs have been implemented in all public acute hospitals. Future actions • All hospitals (both public and private) were required to implement ASPs, including the monitoring of AMU, through licensing, accreditation, and quality assurance frameworks. • ASPs in veterinary medicine to be developed. • Guidelines on the appropriate AMU in community hospitals, ambulatory facilities (e.g. dialysis centres), and primary care clinics to be issued to improve prescribing practices; national guidelines for prudent AMU in livestock and veterinary medicine to be established. Regulations • Prior/ongoing activities • Antimicrobials for human use were classified as prescription only medicines. • Legislation prohibited use of antimicrobials for growth promotion in Singapore farms, and restricted certain antimicrobials for use in feeds and food-producing livestock and aquaculture farms. • Sales of human therapeutic products for veterinary use was restricted to veterinarians, veterinary centres, and farms. • Health Sciences Authority and Agri-Food and Veterinary Authority regulated legislation in human and animal health respectively, while food safety was overseen by Agri-Food and Veterinary Authority and National Environment Agency. Future actions • Veterinary drug registration system and supply chain control covering import, manufacture, distribution, and sale to be set up.	**Policies and guidelines** Prior/ongoing activities • Development of the National Infection Control Guidelines for human health and IPC programmes in hospitals. • Introduction of isolation and selective treatments to farms or pet owners ongoing to prevent and manage diseases in animals; good animal husbandry practices and good aquaculture practices have been developed. • Ongoing Sewerage and Drainage Act for managing antimicrobials discharge in environment, with protocols to ensure high hygiene standards to disinfect sewage and remove antimicrobials to prevent waterborne diseases and contamination by antimicrobials; licensing and audits for Toxic Industrial Waste Collectors. Future actions • Food hygiene programme, education of food handlers to improve hygiene standards, and surveillance of pathogens in food to be improved. **Other strategies** Prior/ongoing activities • Campaigns to promote vaccination; national immunisation policies and guidelines for children, adults, healthcare workers, and travellers; mandatory vaccination for all dogs and cats sold in pet shops; autogenous vaccines in aquaculture; vaccination monitoring and management in farms. • Incentives for good husbandry practices, such as Singapore Quality Egg Scheme for the local layer farms, and Good Aquaculture Practices for Fish Farming.
Thailand	Future actions • Capacity and networking of microbiology laboratories to be strengthened; improvement of epidemiological capacity and networking on AMR planned. • National integrated system of AMR and HAI surveillance and signalling, with alert of emerging pathogens and coordinating systems across agencies at local and national levels to be developed. • AMU in humans, veterinary medicine, and crop production to be measured by using national databases of annual reports of pharmaceutical companies.	**ASP** Future actions • ASP in animal hospitals and clinics, as well as human healthcare facilities, private clinics, and pharmacies to be implemented. • National policy on promoting rational AMU use in hospitals to be developed. **Regulations** Prior/ongoing activities • Animal Feed Quality Control Act allowed only registered drugs to be administered under the supervision of veterinarians. • Ministry of Agriculture and Cooperatives regulated production, sales, and use of medicated feed. Future actions • Antimicrobial distribution control system for both humans and animals to be strengthened. • Promulgation of a legislative order on withdrawal of antimicrobials from a household remedy list and restriction of antimicrobials, especially those at high risk of developing resistance, to be prescription only. • Law enforcement to be enhanced along with social measures to mitigate the problem of inappropriate distribution of antimicrobials.	**Policies and guidelines** Future actions • Efficient systems in healthcare facilities aimed at reducing infection rate and expenses caused by AMR pathogens for human health to be established; measures and systems for management of patients infected by resistant microbes to be developed.
Vietnam	Future actions • National AMR reference laboratory to be established; laboratory capacity, human resources, and equipment to be enhanced; guidelines and standardised test methods to be developed. • National surveillance system on AMU and AMR to be strengthened; system of monitoring and reporting data on hospital infections from hospitals under central authority; warning system to warn about the dangers of AMR to be established. • Monitoring system of safe appropriate AMU in health facilities, livestock, poultry, aquaculture, and cultivation to be established. • Antibiotic residues in foods to be tracked and monitored. • A network to monitoring AMU and AMR in 30 laboratories across the country to be set up.	**ASP** Future actions • Treatment guidelines for medical staff and community, and list of permitted antimicrobials in livestock, poultry, aquaculture, and cultivation to be developed. • No mention of ASPs or incentives for appropriate AMU. **Regulations** Future actions • Compliance to treatment guidelines health facilities to be evaluated; guidelines on AMU in livestock, poultry, aquaculture, and cultivation to be improved. • Comprehensive management of drug quality during the entire process of production, export, import, distribution to prevent poor quality and counterfeit drugs from circulating in the market to be ensured. • Investment in production to supply the market with drugs of good quality, reasonable price to be ensured. • The Council of Medicines and Treatment to monitor compliance to regulations.	**Policies and guidelines** Future actions • Legal documents, policies, national technical regulations, and hospital guidelines for human health to be revised. • Organisation of IPC in examination and treatment facilities to be improved.

Prior/ongoing activities = activities which are either executed once or are still in progress at the point of national action plan publication. Future actions = focus areas and plans to be implemented.

AMR = Antimicrobial resistance. PPS = Point prevalence survey. HAI = Hospital-acquired infection. AMU = Antimicrobial use. ASP = Antimicrobial stewardship programme. IPC = Infection prevention and control. FAO = Food and Agriculture Organization.

**Table 5:** Governance area 2 – Implementation tools (part 2).

Country	Education	Research & innovation	International collaboration
Brunei	Prior/ongoing activities • Health Promotion Centre coordinated activities targeted at increasing public awareness on AMR using multiple communication platforms and technologies. Future actions • Public awareness on antimicrobial resistance (AMR), including the World Hand Hygiene Day and World Antibiotic Awareness Week (WAAW) to be conducted. • Concepts of health literacy, psychology, and behavioural economics to be considered for future campaigns to ensure effective knowledge transfer and effect changes in behaviour. • AMR knowledge and infection prevention and control (IPC) best practices of professionals in human, animal, and environment health to be enhanced, through undergraduate and/or postgraduate curriculum, as well as through periodic continuing education (CE) activities. • Best practices for importers, distributers, farmers, animal owners, and veterinarians to be promoted; in-house CE for slaughterhouse operators, farm operators and livestock inspectors to be established.	Future actions • Surveys and impact assessments planned to gauge knowledge and practice on antimicrobial use (AMU) at different levels of society. • Analysis of economic incentives that contribute towards inappropriate AMU to be conducted. • Evidence generated through research on drivers of AMR to guide implementation activities. • No mention of budget for research; or collaboration.	• No mention of international partnership.
Cambodia	Prior/ongoing activities • AMR awareness campaigns already ongoing, such as the WAAW through mass media channels. • Communication materials for advocacy at the community level were distributed to village health and animal health workers, and officials, for further education to clients. Future actions • AMR to be included as pre-service curricula in medicine, nursing, pharmacy, veterinary medicine, and animal, fisheries, and crops sciences to ensure integration of AMR, antimicrobial stewardship programmes (ASPs), rational AMU, and IPC • Capacity building and continuous training to be conducted for staff and professionals who work in laboratories, surveillance, clinical practice guidelines and IPC. • Impact of behaviour change campaigns to be monitored.	Prior/ongoing activities • Some knowledge, attitudes, and practices (KAP) studies on health providers, farmers, and the general public on AMU have been conducted. Future actions • AMR and related issues (surveillance, laboratories, IPC, rational use, sanitation and hygiene, regulation) as an important component of the national health, agriculture, and environmental research agendas to be integrated in the plan. • Research to be conducted to establish correlations between AMR in human, animal, and environment health; qualitative and operational research on KAP on antibiotics of various stakeholders at different levels to support policy frameworks. • Partnerships between stakeholders to strengthen research activities to be improved. • No mention of research and development (R&D) of antimicrobials, diagnostics, or vaccines; or budget for research.	Prior/ongoing activities • Support from World Health Organization (WHO), Food and Agriculture Organization (FAO), and OIE, World Organisation for Animal Health, provided to conduct situational analysis on AMR • Technical and financial support provided by WHO/FAO/OIE, bilateral agencies, development banks, and international and national non-governmental organisations (NGOs) for development of the national action plan (NAP). Future actions • Dialogues with international partners and NGOs on activities and initiatives to support AMR to be conducted. • Partnerships with international academics, schools, and experts for knowledge sharing, capacity building, research, and other initiatives, to be strengthened. • To participate in the human global antibiotic appropriateness point prevalence survey (PPS).
Indonesia	Prior/ongoing activities • AMR awareness campaigns targeted at professional and general public already rolled out. Future actions • Evidence based campaigns including monitoring and evaluation (M&E) component planned. • AMR and IPC to be included in undergraduate, postgraduate, and CE courses for human and animal health, food industry, and agriculture • Training for IPC for various sectors at different levels (policy makers, programme managers, general people, industry leaders, farmers) and AMR surveillance and laboratory techniques for surveillance and clinical staff, and laboratory personnel to be organised. • Sanitation and hygiene including food handling practices in education for school and college students to be included.	Future actions • National policy for research and innovation to be developed. • Sustainable investment in new medicines, diagnostic tools, and vaccines by engaging the public/private sectors to be promoted. • Existing research capacity and funding appropriateness to be assessed. • Research on AMU data in linkage with the resistance profiles reported by AMR surveillance programme to be conducted; cost effectiveness and feasibility of interventions to be conducted. • Drivers of irrational AMU to be assessed through KAP studies. • Multi-stakeholder platform for collaboration on AMR research and innovation and inventory of relevant networks, initiatives, institutions, and experts was to be established.	Prior/ongoing activities • Technical support provided by WHO Indonesia Country Office and WHO-South-East Asia Regional Office for NAP development; • Technical support provided by WHO, FAO and OIE for capacity building in IPC and biosecurity in various sectors. • Sustained national and international partnerships on containment of AMR, surveillance, and innovations. • Ongoing data sharing with the WHO Global AMR Surveillance System (GLASS). Future actions • National surveillance network to be expanded to the regional and international level.
Laos	Prior/ongoing activities • Educational campaigns such as WAAW, already ongoing and will be expanded to IPC. Future actions • AMR information to be disseminated during village meetings, at temples, or during immunisation outreach programmes. • AMR to be incorporated into university and school activities; • training in medical and veterinary schools, and local government livestock office to be conducted to raise awareness on AMU for teachers, medical students, veterinarians and those working with livestock and fisheries; behaviour change to be monitored. • Training course for IPC to be set up; annual refresher training for provincial laboratory staff to be setup to facilitate continuing professional development.	Prior/ongoing activities • Vague plans on development and improving the capability of modern technology for diagnosis and prevention of AMR. No specifics listed about prioritised innovations. Future actions • Research capacity to be enhanced, including interpretation and sharing of surveillance information. • Research on the economic impact of AMR on patients and farmers to be conducted; KAP studies to be carried out to understand the practices. • Plans to raise government commitment for research funding to be developed. • No mention of collaboration.	Prior/ongoing activities • Funding from various international organisations supported the Lao-Oxford-Mahosot Hospital-Wellcome Trust Research Unit; Institut Pasteur du Laos formed with support from Institut Pasteur Paris. • Ongoing data sharing with an Association of Southeast Asian Nations (ASEAN) surveillance network. Future actions • Technical working group (TWG) with support from WHO/FAO/OIE to be established. • Development of livestock management system for veterinary and fishery use to be coordinated with foreign countries.
Malaysia	Future actions • AMR awareness campaigns including WAAW and community empowerment programmes on AMR to be conducted; dissemination through mass media channels. • KAP surveys to be conducted among public and healthcare professionals. • AMR to be included as a core component of professional education for training of healthcare professional; AMR in school extra-curricular activities to promote understand and awareness. • Educational programmes (conferences, seminars, workshops) planned on hygiene and IPC measures in healthcare care settings, animal husbandry and food processing. • CE programmes on AMR, IPC and biosecurity to be established.	Future actions • Research and trials for use of alternative antimicrobials to be established; diagnosis methods to be improved. • Research on drivers of AMR to be used to guide farmers, public, and user of antimicrobials in the animal health sector. • KAP surveys for public, healthcare practitioners, as well as livestock and aquaculture industries to be conducted. • No mention of budget for research; or collaboration.	Future actions • Participation in WHO Integrated Global Survey on Extended Spectrum Beta-Lactamase-producing Escherichia coli using 'One Health' approach, 'The Tricycle Project'. • One Health Surveillance System for AMR to be established, which promotes participation in regional and global networks and sharing of information.
Myanmar	Future actions • AMR and IPC to be included in undergraduate, postgraduate, and CE courses for human and animal health, food industry, and agriculture. • IPC trainings for various sectors at different levels (policy makers, programme managers, general people, industry leaders, farmers) planned; training on AMR surveillance and laboratory techniques for surveillance and clinical staff, and laboratory personnel to be conducted. • A comprehensive evidence-based public communications campaign integrating M&E to be developed. • KAP studies to be conducted for health professionals, famers, pharmacists, and policymakers.	Future actions • National research policy and agenda to be developed to promote sustainable investment in new medicines, diagnostic tools, and vaccines • Analysis of AMU data in linkage with the resistance profiles reported by AMR surveillance programme to be conducted. • Research on policy areas such as regulatory frameworks and enforcement, stakeholder analysis, information management systems planned. • Research to estimate and characterise burden and risk of AMR and AMU in human, animal and food production sectors, barriers, and drivers for prudent AMU practices • KAP surveys to assess prescribing as well as treatment and care-seeking behaviours. • Multi-stakeholder platform for collaboration on AMR research to be set up.	Prior/ongoing activities • WHO engagement to conduct the situational analysis on AMR. • Data submission to the WHO GLASS. Future actions • TWGs for each strategic area to include international experts, including those from WHO/FAO/OIE. Activities include development of surveillance guidelines and IPC programmes. • Communication campaigns to be launched with support from WHO, FAO and relevant NGOs. • Sustained national and international partnerships on containment of AMR, surveillance, and innovations. • Research platform to develop research consortia and establish collaboration with national and international agencies for implementation of strategic research agenda was mentioned • Expansion of national surveillance network to the international level was mentioned.
The Philippines	Future actions • Rational AMU to be included in school curricula, education programs, and CE of health professionals; training for hospital personnel on IPC implementation; training for pharmacists on rational dispensing of antimicrobials, as well as training and education of farmers and veterinarians to be conducted. • AMR education to be promoted at community level.	Prior/ongoing activities • Dedicated research budget assigned. Future actions • Research on development of new antimicrobials and innovative technologies planned. • AMR research agenda for human health to be developed, engaging academic and other research institutions. • AMR detection, prevention, and control to be included in National Unified Health Research Agenda. • No mention of collaboration.	Prior/ongoing activities • Support from WHO-Western Pacific Regional Office was provided to conduct the country situation analysis on AMR. • Participated in Asian Network for Surveillance of Resistant Pathogens, Codex Alimentarius meetings to discuss AMR issues and Asia Pacific Economic Cooperation to design solutions. • Collaborated with Malaysia to conduct a rapid assessment for regulatory measures to combat AMR.
Singapore	Prior/ongoing activities • AMR awareness campaigns already implemented, including WAAW in hospitals and at community level, World Veterinary Day to raise awareness of AMR to local veterinarians, and promotion of infection prevention through vaccination and social hygiene measures. • AMR already included in education curricula of veterinary and other healthcare professional programmes. Future actions • Training programme to be developed to improve the understanding of ASP in the veterinary and farming sector. • CE initiatives for health and veterinary professionals to be strengthened. • Campaigns to be expanded to educate on proper waste disposal with antimicrobials, AMR in animals and infection prevention for pet owners, feed manufacturers, distributors of veterinary drugs, veterinary technicians and para-professionals. • Evaluation of campaigns proposed to assess changes in KAP.	Prior/ongoing activities • Development of new diagnostics and therapeutics through partnerships underway; rapid, on-site test kits to detect animal bacterial pathogens to aid diagnosis and guide appropriate treatment; research into developing viable alternatives for farming industry, such as vaccines, pre- and probiotics and technologies for pathogen control to reduce reliance on antimicrobials. • Funding for AMR research was available. Future actions • Priority research areas to be identified such as transmission pathways, advanced surveillance methods, socio-behavioural research on KAP of AMR and prescription patterns, and socioeconomic impact of AMR. • National research agenda with all stakeholders to be developed; existing research to be mapped and platform for collaboration for multi-disciplinary research in humans, animals, food, and environment sectors to be created.	Prior/ongoing activities • Core strategies were underpinned by the principle of international collaboration. • Provided support to member states of ASEAN to improve health systems by sharing technical expertise and experience; led in strategies for AMR in the livestock sector regionally. • Participated in regional and international research. Examples include the Global Water Research Coalition, global PPS on human AMU and AMR, and projects with Wellcome Trust-funded research units. • Ongoing sharing of relevant surveillance data at the national, regional, and international levels; participated in global surveillance networks such GLASS and OIE global database.
Thailand	Future actions • Relevant stakeholders in livestock and agriculture, IPC personnel to be educated regarding appropriate usage. • Community knowledge and awareness to be raised regarding appropriate usage by partnering civil society and mass media agencies. •	Future actions • Research to be promoted to guide efficient AMR operations. • KAP studies to be conducted every two years to assess public knowledge on AMR and appropriate AMU. • No mention of R&D of antimicrobials, diagnostics, or vaccines; budget for research; or collaboration.	Prior/ongoing activities • Included collaborative role at international level in sub-objective. • `Development of Thailand AMR Strategic Plan' was funded by WHO; Ongoing technical support from FAO to set food standards, understanding the economic impact of limiting AMU in agriculture, and to establish a collaboration framework for technical capacity building, surveillance and risk management of transmissible diseases. • Data sharing with GLASS.
Vietnam	Future actions • Training in microbiology and antibiotics in universities, medical- pharmacy schools and clinical pharmacy practice to be conducted. • Training on technical expertise on clinical microbiology for laboratory staff, AMR surveillance systems and IPC for healthcare workers to be conducted. • Awareness programmes for community, health workers, people who work in the fields of livestock, poultry, aquaculture and cultivation on antibiotics and drug resistance to be implemented. • Communication activities through mass media channels (TV spots, radio spots, knowledge) and seminars planned to educate community on AMR.	Future actions • Scientific research on drug resistant bacteria, hospital infections, as well as AMR in livestock, poultry, aquaculture, and cultivation to be carried out. • Research capacity in central hospitals and universities to be enhanced, particularly for new diagnostics and treatment of infectious diseases, microbiology testing, quality control, microbiology laboratory. • Assessment of community knowledge about AMR to be conducted. • Coordination with relevant agencies to promote research cooperation planned. • No mention of R&D of antimicrobials or vaccines; or budget for research.	Future actions • International cooperation on continuous training and research on drug use, and clinical pharmacy practice emphasised. • Strengthening of international cooperation, experience exchange and sharing, participate in workshops, scientific conferences, and forums mentioned.

Prior/ongoing activities = activities which are either executed once or are still in progress at the point of national action plan publication. Future actions = focus areas and plans to be implemented.

AMR = Antimicrobial resistance. IPC = Infection prevention and control. CE = Continuing education. WAAW= World Antibiotic Awareness Week. AMU = Antimicrobial use. ASP = Antimicrobial stewardship programme. KAP = Knowledge, attitudes, and practices. R&D = Research and development. WHO = World Health Organization. FAO = Food and Agriculture Organization. NGO = Non-governmental organisation. NAP = National action plan. PPS = Point prevalence survey. M&E = Monitoring and evaluation. GLASS = Global AMR Surveillance System. ASEAN = Association of Southeast Asian Nations. TWG = Technical working group.

##### 3.2.2.1 Surveillance

Surveillance involves ongoing systematic data collection, analysis, and interpretation to plan, conduct, and evaluate policies. Surveillance for AMU and resistant organisms in both human and animal health, supported by adequate laboratory capacity and capability, were thoroughly discussed in all NAPs. However, AMU appropriateness was not as frequently stated. In addition, AMR surveillance was mainly focused on human and animal health, and less so on the environment, as only three countries highlighted environmental AMR surveillance. Five NAPs had existing surveillance systems in public hospitals and were to be extended to private hospitals and community healthcare facilities. Nationwide laboratory capacity strengthening for efficient national surveillance of resistant organisms were also emphasised.

Other than Brunei, Singapore and the Philippines, all other countries mentioned developing alert mechanisms for early detection and reporting of newly emerged resistance. HAI surveillance was highlighted by all except Laos. Surveillance of food products for antibiotic residues was distinctly emphasised in Singapore's NAP while Indonesia, Myanmar, and the Philippines alluded to it. Perhaps, this emphasis could be attributed to more than 90% of Singapore's food being imported. All countries mentioned regional and national level surveillance networks, and integration for better information sharing.

##### 3.2.2.2 Optimising antimicrobial usage

Antimicrobial stewardship programmes (ASPs), defined as coordinated interventions to improve appropriate AMU by promoting optimal drug regimen selection [42], was mentioned by all countries except Vietnam. Most countries discussed ASP in both human and animal health, except Cambodia and Malaysia which focused on human health only. Treatment guidelines and AMU restriction policies help encourage appropriate AMU for human and animal health. This was accounted for by all countries except Thailand. Incentives or penalties to reduce inappropriate AMU in general (Brunei), in human health (Singapore and the Philippines), and in animal health (Malaysia), were mentioned, but no detailed plans were presented. Rapid diagnostic tools to support ASP initiatives was briefly mentioned by Singapore and rarely discussed by others.

Medicines regulations including laws, accreditation, or financial penalties to reduce inappropriate AMU were extensively discussed in all NAPs, especially in human and animal health. Examples include classification of antimicrobials as prescription only medicine that can only be prescribed by licensed professionals, regular audits on antimicrobial sales in community pharmacies, regulations on AMU promotion and marketing, as well as systems for AMU documentation. These regulations also aimed to prevent poor quality antimicrobials from circulating in the market. Presence of enforcement agencies to support regulations was mentioned in nine NAPs. However, none stated a dedicated budget to support legislation monitoring and enforcement. Regulations in the environmental sector were only mentioned by Cambodia.

##### 3.2.2.3 Infection prevention and control

Poor sanitation and hygiene measures can spread drug-resistant infections. IPC aims to reduce transmission of resistant organisms and minimise infection risks. IPC policies and guidelines across human and animal health were widely discussed across NAPs. For human health, IPC strategies included setting up dedicated committees in hospitals, developing policies on hand hygiene, and management of patients with multidrug-resistant organisms. Only Singapore mentioned ongoing regular testing of drinking water. For animal health, good agricultural practices and biosecurity improvements were recommended to prevent diseases. Environmental IPC was described by Cambodia, Indonesia, Lao, Myanmar, and Singapore. Examples included waste management systems in hospitals and farms for safe disposal of unused or expired antimicrobials and effluent treatments. Lastly, IPC for food products was only mentioned by Cambodia, Myanmar, the Philippines, and Singapore. Examples included programmes to improve sanitation and hygiene through education on food handling practices, and food pathogens surveillance.

Brunei, Cambodia, Indonesia, Malaysia, Myanmar, and Singapore mentioned other strategies to minimise infection risk, such as immunisation programmes for both human and animal health. Incentives or penalties for IPC policies, however, were rarely mentioned, and only featured as facilitation of market access for products from farms with audit certifications in Malaysia and Singapore.

##### 3.2.2.4 Education

Public awareness and education on AMR are essential to promote rational prescribing practices and reduce non-judicious AMU. Certification or programmes to ensure a basic education for all involved professionals were widely discussed across all NAPs. Except for Thailand, all NAPs focused on inclusion of AMR and AMU in pre-service curricula of healthcare and veterinary professionals. Continuing education programmes, including on-the-job training updates, workshops, and conferences, to expand knowledge and sustain efforts to tackle AMR, were highlighted by all countries except Thailand.

Public awareness campaigns and educational programmes, including the World Antibiotic Awareness Week, featured in all NAPs. While many campaigns were already rolled out across mass media channels, only Indonesia and Myanmar mentioned evidence-based behaviour change campaigns being planned. Efforts for ongoing, sustained public education were rarely mentioned. All NAPs foregrounded the need to monitor knowledge transfer and behaviour change, with the Philippines being an exception. However, details were absent.

##### 3.2.2.5 Research and innovation

Innovation was featured in all NAPs as R&D of novel therapeutics and diagnostics, and vaccines briefly, except for Cambodia, Thailand, and Vietnam. Indonesia also included a component on innovative financing. Singapore provided specific R&D examples including on-site test kits for detection of animal bacterial pathogens for diagnosis and treatment. All NAPs emphasised on research to understand drivers and impact of AMR. Research to understand knowledge, attitudes, and practices of stakeholders and socioeconomic impact of AMR was described as well. It was touted as a tool for AMR advocacy and development of targeted interventions such as economic incentives or disincentives for reducing inappropriate AMU. Encouraging research collaborations through development of multi-stakeholder national research agenda across disciplines was briefly mentioned in five NAPs.

##### 3.2.2.6 International collaboration

Due to complex, interrelated drivers of AMR, no nation can combat AMR alone. International collaboration is imperative in tackling this global health crisis. Partnerships as well as donor and development partner engagement were discussed by all countries, except Brunei. Funding, technical support, and dialogues for initiatives to support AMR were highlighted too. Singapore led and supported these efforts both locally and regionally. Regional and international research consortia to support knowledge generation and innovation were discussed. Eight countries reported data to international databases including the WHO global AMR surveillance system (GLASS).

#### 3.2.3 Monitoring and evaluation

M&E is key in determining effectiveness of a plan and providing evidence to inform policies. This must be accompanied by information dissemination and reports. These domains are presented in Table 6.

**Table 6:** Governance area 3 – Monitoring and evaluation.

Country	Effectiveness	Feedback mechanism	Reporting
Brunei	Prior/ongoing activities • Monitoring and evaluation (M&E) through data trends, the distribution and use of antimicrobials as well as compliance to antimicrobial stewardship programmes (ASPs) and national antibiotic guidelines. Future actions • Periodic assessments of policies, interventions and good practices implemented in human, animal, and environmental health to ensure that they achieve their intended purposes.	Future actions • Sector specific surveillance programmes to produce data trends, that can be analysed and communicated within respective centres, to be implemented. This will help to inform policy decisions and review processes.	Prior/ongoing activities • Data collection on human, animal health, and aquaculture was reported in accordance with standards set by World Health Organization (WHO) and OIE, World Organisation for Animal Health. Future actions • Reporting of priority pathogens according to 'Global priority list of antibiotic-resistant bacteria to guide research, discovery, and development of new antibiotics' to be expanded to all public and private healthcare settings • Reporting of drug residue limits and antimicrobial resistance (AMR) surveillance programme testing to be done by programme owners.
Cambodia	Prior/ongoing activities • Necessity of M&E of inputs, processes, outcomes, protocols, staff training, and drug quality acknowledged in the plan. • Two types of M&E mentioned: (1) Ongoing annual routine data collection and (2) special surveys and evaluation activities mid-term and end-of-term. • Baseline values of the proposed indicators to be collected. Future actions • Routine data collection and reporting to use indicators and reporting mechanisms to be built into normative programmes and departments, such as AMR surveillance in laboratories, ASPs, and infection prevention and control (IPC).	Future actions • Existing good practices to be assessed, reviewed, and translated into new programmes for AMR for animal production, veterinary and agriculture practitioners. • Evidence to be used for AMR related advocacy for policy support.	Prior/ongoing activities • Publication of research findings on AMR detection and genetic characterisation of resistant bacteria; sharing of information in regional and international conferences and working group meetings. Future actions • Reporting and information sharing between human health, agriculture, and environment laboratories at national level to be harmonised. • Monitoring data on IPC and other activities to be reported.
Indonesia	Prior/ongoing activities • Key indicators for each objective included for each intervention. Future actions • Each technical working group (TWG) to be responsible for M&E of plans in their individual strategic objectives and to develop appropriate recommendations. • Baseline surveys to be conducted to assess the extent, barriers, and enablers of ASPs. • Evaluation of pilot campaigns; evidence-based campaigns designed with M&E component.	Prior/ongoing activities • Flexibility was built into planning process including monitoring and reporting arrangements, to allow for determination of priority actions required. Future actions • Behaviour change campaigns to use evidence generated for designing accurate and relevant messages targeting priority groups.	Future actions • Status of implementations to be reported to the Inter-Ministerial Steering Committee, national agencies, and international partners. • Integrated human and animal IT platform for regular AMR and hospital-acquired infection (HAI) surveillance reporting to be developed. • Indicators for national surveillance system to include data reports from surveillance sites, timeliness and completeness of surveillance reports, and assessment reports of performance of National AMR surveillance programme. • Tracking and reporting of drug quality control planned.
Laos	Future actions • Overall AMR programme to be evaluated against international standards defined by WHO using the ' Joint External Evaluation tool' and ‘M&E framework of the Global Action Plan on AMR'. • The progress of each strategic objective to be evaluated before and after implementation; evaluation indicators included for each objective. • No mention of ASP evaluation.	• No mention of feedback mechanism that informs the system.	Prior/ongoing activities • Lao-Oxford-Mahosot Hospital-Wellcome Trust Research Unit collects data on AMR on a daily basis and publishes the data monthly. • Reporting to the Ministry of Health, hospitals, the health sector, and a surveillance network of the Association of Southeast Asian Nations (ASEAN) was mentioned. • Reports on AMR surveillance in the veterinary, livestock and fishery sector were mentioned. Future actions • AMR data/burden to be collected and published in national AMR report.
Malaysia	Prior/ongoing activities • Monitoring of antimicrobial use (AMU) and pattern in healthcare facilities • Ongoing monitoring for veterinary drug residues from the aquaculture farm • Evaluation indices for each objective were listed after each intervention. Future actions • National Coordinating Centre on AMR to monitor activities and report to the National AMR Committee. • M&E to be conducted periodically to evaluate the effectiveness and impact of the national action plan (NAP).	• No mention of feedback mechanism that informs the system.	Future actions • Reporting of antibiotic consumption sales data planned. • Reporting system in Ministry of Health and university hospitals to be improved. • Comprehensive integrated AMR surveillance report to be developed. • AMU report as a condition for license application or renewal; report of AMU surveillance in veterinary, fish and shrimp aquaculture. • Participation in joint conferences, symposiums, and scientific meetings planned.
Myanmar	Prior/ongoing activities • Illustrative indicators for each objective included for each intervention. Future actions • Each TWGs responsible for M&E of plans in their individual strategic objectives and develop a set of workable recommendations; • Illustrative indicators for each objective included for each intervention. • Mechanisms to be established to monitor AMU on a national scale to inform interventions.	Prior/ongoing activities • Flexibility was built into planning process including monitoring and reporting arrangements, to allow for determination of priority actions required in a stepwise manner. Future actions • Behaviour change campaigns to use evidence generated for designing accurate and relevant messages targeting priority groups.	Future actions • Status of implementations to be reported to the Multi-Sectoral Steering Committee, national agencies, and international partners. • Develop an integrated human and animal IT platform for regular AMR and HAI surveillance reporting; regular data of AMR and resistance profiles of priority pathogens for human, animal and food to be made available to the AMR Surveillance Coordination Unit. • Disseminate research evidence to inform national and local policies and strategic interventions in different strategic objectives to reduce the need for antimicrobial in different settings. • Tracking and reporting of drug quality control planned.
The Philippines	Prior/ongoing activities • Collaborated with Malaysia for rapid assessment of regulatory measures. Future actions • Surveillance and monitoring system for AMR and AMU in food-producing animals to be developed.	Future actions • Systems to be developed for reporting and feedback of surveillance data, and for translating findings to concrete government policies and programmes.	Future actions • IPC monitoring reports from all hospitals to be published. • Report on the quality of registered antimicrobials and the presence of unregistered antimicrobials to be made available. • Reports on AMR in animal species, banned drugs and annual integrated report on AMU, AMR, and HAI, to be published regularly.
Singapore	Prior/ongoing activities • Monitoring of IPC indicators to determine effectiveness of measures • Monitoring of vaccine uptake and measures to encourage vaccination being implemented. Future actions • Evaluation of ASPs in hospitals to be conducted to enhance effectiveness and to develop guidelines based on identified good practices • Goals for the next five years and M&E indicators to be detailed subsequently. • Surveillance information to be used to drive impact assessment of interventions, guide policy development, and develop appropriate education and mitigation measures.	Future actions • Surveillance and risk assessment to drive impact assessment of practices on resistance development, monitor effectiveness of interventions, guide policies for AMU in food animals, and guide appropriate education and mitigation measures.	Prior/ongoing activities • Reporting of national aggregate data on AMR; data reporting for priority pathogens across sectors. • Reporting of AMR and IPC indicators by all hospitals mandated through licensing, accreditation, and quality assurance frameworks • AMU with appropriateness and ASP intervention acceptance rates were reported bi-yearly. Future actions • Relevant surveillance data to be published and reported at the national, regional, and international levels.
Thailand	Future actions • Aimed for ASP M&E in healthcare facilities. • M&E to be based on the developmental evaluation approach, including the measurement of progress towards defined milestones, targets, and goals in reference to an established baseline.	Future actions • Evidence from M&E processes to guide effective NAP implementation.	Prior/ongoing activities • Annual reports of pharmaceutical production and importation on antimicrobial consumption in humans and veterinary medicine production shared with Food and Drug Administration. • Report on 'The Landscape of AMR situation and actions in Thailand' was a common source of information for AMR stakeholders and public. Future actions • The AMR surveillance system provides timely reports on the AMR epidemiological situation in both humans and animals.
Vietnam	Future actions • Monitoring system of drug resistance was not in place yet. • Evaluation indicators, a system for collecting and processing information, and websites for tracking, M&E of AMR to be developed. • Monitoring indicators for IPC and HAI in hospitals to be developed. • Indicators for AMU and treatment compliance in hospitals and community to be developed. • Survey and assessment of the community's knowledge about drug resistance planned.	• No mention of feedback mechanism that informs the system.	Future actions • Monitoring systems and reporting data on hospital infections to be established. • Database of national infection control to be created. • Conferences to be organised to disseminate and educate laws on the prevention of drug resistance.

Prior/ongoing activities = activities which are either executed once or are still in progress at the point of national action plan publication. Future actions = focus areas and plans to be implemented.

M&E = Monitoring and evaluation. ASP = Antimicrobial stewardship programme. WHO = World Health Organization. AMR = Antimicrobial resistance. IPC = Infection prevention and control. TWG = Technical working group. HAI = Hospital-acquired infection. AMU = Antimicrobial use. NAP = National action plan.

##### 3.2.3.1 Effectiveness

M&E was mentioned in all NAPs to various degrees. Eight NAPs with integrated M&E plans listed evaluation indices for each intervention. Brunei and Singapore mentioned it briefly, highlighting that full indicators will be detailed in subsequent plans. Some countries have nominated agencies and TWGs to oversee M&E processes.

##### 3.2.3.2 Feedback mechanisms

Feedback mechanisms to relay data to governing bodies were missing from the NAPs of Laos, Malaysia, and Vietnam. Others briefly discussed measures in place, including regular review and updating to translate findings into concrete evidence-based government policies and programmes.

##### 3.2.3.3 Reporting

Data reporting was mentioned by all NAPs. Reports for AMR and AMU surveillance and implementation status were highlighted. However, frequency of reports was sporadic and reporting authority was unclear. Data dissemination through publications and conferences at the national, regional, and international levels was also discussed.

#### 3.2.4 Sustainability

Assessment of resource needs and dedicated budgets are paramount for effective NAP implementation. Together with M&E activities, considerations for future budget requirements allow for expansion of implementation plans in a sustainable manner. Domains related to sustainability are presented in Table 7.

**Table 7:** Governance area 4 – Sustainability.

Country	Fund and resource allocation	Future expansion of implementation plans
Brunei	Future actions • Detailed operational plans to support the national action plan (NAP), including funding for the next five years to be detailed in subsequent NAP.	• Incremental approach to be adopted to continuously improve and scale up existing activities in the NAP and ensure economic sustainability of programmes. Examples included scaling up of existing facility level hand hygiene programmes and antimicrobial stewardship programmes (ASPs).
Cambodia	Future actions • Assessment on costing for budget planning to be conducted and sustainable funding mechanisms to be identified. • Regular review of funding resources planned. • Political and financial support for antimicrobial resistance (AMR) initiatives to be ensured through advocacy. • Planning and costing of workforce skills-mix to identify capacity needs to be conducted; building human capacity and availability of professionals/staff planned.	• No mention of future expansion of implementation plans.
Indonesia	Future actions • Operational, budgetary and monitoring and evaluation (M&E) plans to be finalised before rolling out AMR activities. • Assessment of resources of every centre for AMR and antimicrobial use (AMU) surveillance to be performed. • Resources required including staffing, expertise and finance were listed for certain interventions in the NAP. • Human resources, materials, and funding for research to be prioritised.	• Phased implementation of interventions to be done, consisting of five stages adapted from the Indicator Standards Assessment Tool developed by UNAIDS, with plans for sustainability. Examples included roll out of AMR awareness campaigns and animal vaccination campaigns on a limited scaled with a plan for phased nationwide scale up.
Laos	Future actions • Government commitment for funding to support AMR surveillance and research to be raised • Resource allocation to support the national strategic plan to be ensured. • Human resource and funding for infection prevention and control (IPC) to be improved in government hospital wards.	• No mention of future expansion of implementation plans.
Malaysia	Future actions • Assessment of health burden caused by AMR to be conducted to guide policymakers in identifying priorities and appropriate budget allocation. • Number of IPC personnel, and other healthcare professionals to be increased through training and certification. • Personnel such as infection control nurses in critical care units, as well as clinical microbiologists and ASP pharmacists, to be placed at required sites of in all major specialist hospitals.	• No mention of future expansion of implementation plans.
Myanmar	Future actions • Operational, budgetary, and M&E plans for successful implementation of the activities to be finalised. • Detailed planning along with the budget allotted for the respective activities to be done by national stakeholders. • Assessment of resources of every centre for AMR and AMU surveillance to be conducted. • Research capabilities to be assessed to develop plans to prioritise human resources, materials, and funding for research.	• Phased implementation of interventions to be done, consisting of five stages adapted from the Indicator Standards Assessment Tool developed by UNAIDS, with plans for sustainability. An example included roll out of AMR awareness campaigns on a limited scaled with a plan for phased nationwide scale up.
The Philippines	Prior/ongoing activities • Fund allocation for research was listed. Future actions • Incentives and research funding for innovators to be provided • Funding requirements to be incorporated in the annual budget proposals of the respective member-agencies. • Possible fund sources to be identified. • Costs and potential savings from AMR interventions to be assessed.	• No mention of future expansion of implementation plans.
Singapore	Prior/ongoing activities • Funding being provided to support ASPs in all public acute hospitals, and • Multiple funding streams were available for AMR related research • AMR recognised as one of the top three research focus areas under government funded grant. Future actions • Dedicated funding for inter-agency collaborative AMR research that fits the national AMR research agenda. • Goals for the next five years, including funding priorities to be detailed subsequently.	• Expansion of AMR surveillance programme to include private hospitals and the community setting, all animal production sectors, as well as aquaculture. • Initiatives to roll out education efforts to promote prudent AMU in livestock and infection prevention.
Thailand	Future actions • Stimulation of political commitment for allocation of appropriate resources to address AMR issues effectively and sustainably proposed.	• Initiatives to roll out implementation plans in a small sample of sites, with plans for expansion was discussed. An example included ASPs in two animal hospitals as a starting point for system development to promote ASP in animal hospitals.
Vietnam	Future actions • Investment from state budget and other legitimate funds to implement the NAP was mentioned. • Resources from international and/or non-governmental organisations to be mobilised. • Investment in infrastructure, support facilities and equipment to be increased to meet the demands of IPC, microbiology testing, AMR monitoring, and drug quality control.	• No mention of expansion of implementation plans.

Prior/ongoing activities = activities which are either executed once or are still in progress at the point of national action plan publication. Future actions = focus areas and plans to be implemented.

NAP = National action plan. ASP = Antimicrobial stewardship programme. AMR = Antimicrobial resistance. M&E = Monitoring and evaluation. AMU = Antimicrobial use. IPC = Infection prevention and control.

##### 3.2.4.1 Funding and resource allocation

If political stakeholders are aware and privy to the funding needs for AMR activities, they can help in allocating adequate budgets. All NAPs acknowledged that funding and resources were required to roll out their plans by priority allocation for AMR research, surveillance, and ASPs. Although, only Philippines shared budgets allocated for AMR activities. Strategies for mobilising resources and securing adequate funds for AMR activities were highlighted by five countries. Brunei and Singapore mentioned that detailed plans will be covered in the subsequent NAPs. Four NAPs mentioned the need for regular resource assessment to assist management of budget allocation and priorities. Other commonly discussed resources included human resources of manpower and skills, as well as facilities and infrastructure for effective implementation.

##### 3.2.4.2 Expansion plans

Five NAPs mentioned incremental scaling of implementation plans and operations with future sustainability through continuous improvement. This was most prominent in NAPs of Indonesia and Myanmar, which discussed five implementation phases, each to be informed by the experience from previous phase.

#### 3.2.5 One Health engagement

One Health engagement, highlighted in WHO's situational analysis tool as activities against AMR engaging two or more organisations that represent human health, animal health, and environmental sectors, was not distinctly underpinned in Anderson's framework [28]. However, drawing from GAP, its principle was present across all other governance areas, with involvement of all three sectors. As such, One Health engagement should be a separate governance area, influencing the other four areas. While all NAPs were designed using a One Health approach, clarity of achievable goals and future plans more frequently involved human and animal health sectors as compared to the environmental sector.

## 4 Discussion

Our study assessed ten AMR NAPs from the SEA countries, using a governance framework adapted from the one published by Anderson [27]. As strategic tools for AMR containment, NAPs are a result of political momentum facilitated by coordinated efforts of WHO [7]. The NAPs aimed to serve as guides for key stakeholders, providing integrated responses against AMR through identification of priority areas for work and collaboration. These NAPs were well-aligned with the GAP, although international guidance on specific topics such as the ‘WHO guidelines on use of medically important antimicrobials in food-producing animals’ were not mentioned, possibly due to the broad nature of NAPs [43].

We identified some unique features of these NAPs as well. Local AMR burden was elaborated in majority of NAPs. Thailand even described the NAP development and participatory process providing a detailed chronology on actions taken. These features reinforced transparency, an enabling factor for trust and efficient collaboration. Although NAPs have described some ongoing activities and their future plans across the document, Brunei and Singapore have neatly defined sections on ongoing and future actions contained within each main objective. This provided a clearer understanding of the implementation state at time of publication.

Overall, there were a few weak areas that surfaced across NAPs, including accountability, sustained engagement, equity, behavioural economics, sustainability plans and transparency, international collaboration, and integration of environmental sector.

Accountability, an integral feature of good governance, projects a sense of ownership onto individuals or institutions, and obligates answerability to other actors about their decisions, actions, and results, thereby facilitating collaboration for cross-cutting problems [4,44]. Unfortunately, it was rarely detailed in NAPs and implications of unmet objectives were absent. Such sanctions without enforcements can diminish accountability, and inadvertently threaten public trust in agencies [45]. True success against AMR is difficult to quantify, as targets have tenuous or indirect linkage with improvement of health and well-being of a population. Surrogate indicators such as percentage of AMU were often used instead, and each country determined those based on their context. However, absence of clearly defined goals with clear explanation of roles and responsibilities in some NAPs, may have weakened accountability. Participation during NAP development was emphasised in the majority, but fewer discussed sustained engagement. In addition, most forms of public engagement only involved a top-down approach. Stakeholder participation, along with consistent community engagement is necessary for sustainable and effective policy implementation [7]. For example, Thailand held a health assembly involving representatives from government, academia, private sector, and civil society from all 77 provinces of Thailand [46]. Representatives drafted resolutions discussed at provincial forums and brought them up for discussion during the assembly.

Considerations for various exposure levels and vulnerabilities to AMR should be integrated in NAPs to ensure no one is left behind. For instance, a report from WHO highlighted that women from low-income families or lower educational levels were more vulnerable to certain infections and are less likely to afford first- and second-line treatments [47]. Even though accessibility, affordability, and socio-economic aspects of equity were accounted for, gender considerations were not mentioned at all in the NAPs.

Influencing human behaviour regarding non-judicious AMU is complex, requiring incorporation of knowledge, attitude, practices, culture, psychology, and behavioural economics. Interdisciplinary research is required to understand the interplay of these factors and how they influence AMU. While the need of monitoring knowledge, attitude, and practices among professional and public was strongly underpinned across NAPs, only Indonesia and Myanmar mentioned translating evidence to inform future behaviour change campaigns. Sustained efforts need to be channelled towards designing behavioural change campaigns and evaluating them to inform future initiatives.

Without proper assessment of funding, existing resources and planning, stakeholders might have restricted information to make best policy decisions. If necessary, AMR can be an addition to other priorities, in situations where exclusive priority to AMR is not possible [23]. Although funding and resource allocation were described by all countries, only the Philippines shared budget allocated for AMR activities. Transparency, and accountability of funding and resource allocation have been suggested to be useful; a study reported that civil society organisations' access to and analysis of government budgets and expenditure data enabled them to monitor budgetary conformance with key regulations and led to better resource usage [48]. This must be coupled with flexibility to determine incremental M&E targets and priority actions based on current resources in a stepwise manner [7].

No nation can tackle AMR on its own. Channels of international collaboration can help reduce activity duplication, strengthen ongoing AMR work, and draw lessons from country experiences. Most countries were enrolled in GLASS, enabling AMR surveillance data sharing worldwide. However, active engagement was rarely alluded to. International collaborations highlighted were primarily donor or development partner engagements, not applicable to high-income countries. Options for achieving global collective action against AMR have been discussed, including establishment of international multi-stakeholder partnership, working groups and advocacy [4]. Call-to-action declarations and workshops will be necessary to promote a shared vision that provides leadership and consensus building among stakeholders for plans to address AMR [23].

Although the One Health approach was highlighted by all NAPs, plans on the environmental sector, however, were still inadequate. This was especially so for environmental AMR research and surveillance, education on environmental impact of AMU, as well as IPC policies on safe effluent disposal and legislation that regulates these policies. Environmental health, reported to be underrepresented in One Health networks, is an important concern as ecosystems may contain reservoirs of resistant organisms, potentially threatening human and animal populations [49]. There is a need for better integration of environmental issues with the rest of the plans. One Health approach is an effective way of tackling cross-cutting, complex problems like AMR. However, challenges in implementing One Health strategies are multi-fold. Evidence suggests that struggles of collaboration and promoting equal participation across domains, conflicts of interest of multiple collaborators, coordination, and inadequate M&E deter the efficiency of these strategies [50]. Therefore, understanding the complexity of One Health initiatives is imperative to design and implement these strategies in a more successful and innovative way.

While legally binding treaties or mandates have been considered the gold standard to achieve a social goal [51,52], in the AMR context, solutions may need to be compatible with local needs, context, and culture. For instance, although enforcing prescription-only laws to limit over-the-counter antimicrobial sales may seem a viable solution, it may reduce access to antimicrobials particularly in resource-constrained settings. Therefore, AMR containment requires finding strategic balance between preventing irrational AMU, while ensuring equitable access to these lifesaving drugs. Regulations instituted through a combination of non-binding such as political declaration and binding agreements at multiple levels and across different sectors are more likely to be effective.

As the COVID-19 pandemic has hijacked global attention and claimed national resources, it might be difficult to secure funds for AMR activities. Furthermore, uncertainty regarding the COVID-19 infection may have augmented the AMR burden. Difficulty in differentiating bacterial from viral pneumonia, coupled with lack of effective antiviral agents as well as high workload and stress levels on healthcare workers may have led to unnecessary antibiotic prescriptions [53]. This could deplete the global antibiotic supply chain, potentially leading to a shortage for those who need them. On a larger scale, excessive AMU due to the pandemic will exacerbate the AMR problem worldwide. Therefore, the impact of COVID-19 on AMR must be considered during NAP implementation henceforth. Some examples include reinforcing ASPs in hospitals, updating clinical treatment guidelines to limit unnecessary AMU, maintaining routine AMR surveillance to detect emerging resistance early, and maintaining immunisation programmes during the pandemic [54].

To our knowledge, this is one of the first studies assessing NAPs using a structured governance framework, modified from Anderson's framework for better adaption for this study [27]. The objective NAP assessment provided an overview of progress made on AMR containment in the SEA region. The framework allowed a structured format of analysis for a comparison of specific areas of interest across NAPs. Notwithstanding the need for context-specific plans, comparison allows for benchmarking and identification of best practices in SEA to further improve strategies. However, our objective NAP assessment might have missed certain measures which existed locally might but were not clearly highlighted [27]. We have minimised this risk by having two independent coders with subsequent discussions to ensure that major points are being represented. A strategic and planned approach to translate evidence-informed policies into practice has been recognised as an important component in health promotion [55]. However, as our study is a desk review, we could only report based on what was published in the NAP. There are aspects which are not achievable, including: (1) understanding policy processes and discussions that stakeholders had during NAP development; (2) assessing translation of NAPs to actual implementation on the ground; and (3) exploring whether binding or non-binding regulations are more effective in SEA. To elucidate these aspects, the next phase of our research project will involve discussions with stakeholders through in-depth interviews, and stakeholder analyses to explore impact of their roles and social networks on AMR policies.

## 5 Conclusion

Using a governance framework, this paper has analysed and categorised the content of NAPs from SEA into five governance areas. By assessing NAPs and comparing governance strategies we were able to identify policy priorities, useful features of certain NAPs, and specific areas that should be strengthened, including accountability, sustained engagement, equity, behavioural economics, sustainability plans and transparency, international collaboration, as well as integration of environmental sector. Enhancement of these areas and adoption of best practices will drive improved policy formulation and its translation into effective implementation.

## Declaration of Competing Interest

Dr. Hsu reports grants from CoSTAR-HS Collaborative Centre Grant from the National Medical Research Council, Singapore (NMRC/CG/C005/2017); Dr. Legido-Quigley reports grants from SPHERiC Collaborative Centre Grant from the National Medical Research Council, Singapore (NMRC/CG/C026/2017_NUHS), during the conduct of the study.
